# Factors associated with HIV positive sero-status among exposed infants attending care at health facilities: a cross sectional study in rural Uganda

**DOI:** 10.1186/s12889-018-5024-6

**Published:** 2018-01-16

**Authors:** Methuselah Muhindo Kahungu, Julius Kiwanuka, Frank Kaharuza, Rhoda K. Wanyenze

**Affiliations:** Makerere University School of Public Health, P.O. Box 7072, Kampala, Uganda

## Abstract

**Background:**

East and South Africa contributes 59% of all pediatric HIV infections globally. In Uganda, HIV prevalence among HIV exposed infants was estimated at 5.3% in 2014. Understanding the remaining bottlenecks to elimination of mother-to-child transmission (eMTCT) is critical to accelerating efforts towards eMTCT. This study determined factors associated with HIV positive sero-status among exposed infants attending mother-baby care clinics in rural Kasese so as to inform enhancement of interventions to further reduce MTCT.

**Methods:**

This was a cross-sectional mixed methods study. Quantitative data was derived from routine service data from the mother’s HIV care card and exposed infant clinical chart. Key informant interviews were conducted with health workers and in-depth interviews with HIV infected mothers. Quantitative data was analyzed using Stata version 12. Logistic regression was used to determine factors associated with HIV sero-status. Latent content analysis was used to analyse qualitative data.

**Results:**

Overall, 32 of the 493 exposed infants (6.5%) were HIV infected. Infants who did not receive ART prophylaxis at birth (AOR = 4.9, 95% CI: 1.901–13.051, p=0.001) and those delivered outside of a health facility (AOR = 5.1, 95% CI: 1.038 – 24.742, *p *= 0.045) were five times more likely to be HIV infected than those who received prophylaxis and those delivered in health facilities, respectively. Based on the qualitative findings, health system factors affecting eMTCT were long waiting time, understaffing, weak community follow up system, stock outs of Neverapine syrup and lack of HIV testing kits.

**Conclusion:**

Increasing facility based deliveries and addressing underlying health system challenges related to staffing and availability of the required commodities may further accelerate eMTCT.

## Background

Sub-Saharan Africa contributes 25.6 million people living with HIV out of the 36.7 million people globally [1]. Mother-to-child transmission (MTCT) of HIV accounts for 14% of all new HIV infections worldwide, and may occur during pregnancy, labor and delivery or breastfeeding [2, 3]. Of the estimated 1,800,000 children 0-14 years living with HIV in 2015, 59% lived in East and South Africa [1]. Without intervention, the risk of transmission is 15−30% in non-breastfeeding populations. Breastfeeding by an infected mother increases the risk for an overall transmission by 5-20% [2, 4]. In April 2012 WHO recommended option B+ entailing providing lifelong antiretroviral therapy (ART) to all pregnant and breastfeeding women living with HIV regardless of CD4 cell count or WHO clinical stage [3, 5–7].

According to the 2011 Uganda AIDS Indicator Survey (UAIS), the proportion of women aged 15-49 that were HIV positive increased from 7.5% in 2007 to 8.3% in 2011. HIV infected children under five years were 0.7%; HIV prevalence was higher among children of widows (3%), those whose mothers were HIV-positive (8%) and those whose mothers were dead 4% [8]. To address this high HIV prevalence among exposed infants, Uganda Ministry of Health in April 2012 adopted the WHO revised guidelines to provide lifelong ART to all HIV infected pregnant women irrespective of their CD4 cell count and WHO clinical stage. Infants born to HIV positive mothers start NVP within 72 hours of birth and continue for 6 weeks while the mother is on lifelong HAART [7, 9]. While there is progress in HIV treatment coverage for HIV infected pregnant and lactating women, prophylaxis for the exposed infants and early infant diagnosis have lagged behind. Access to infants’ HIV services is affected by low health facility deliveries, estimated at 57% in 2014 [10, 11]. The 2014 Uganda HIV and AIDS progress report estimated that 5.3% of HIV exposed infants were HIV positive, slightly higher than the elimination target of 5% [9, 11]. However, this prevalence could be an under estimate since it was based on the 1^st^ PCR results for those that accessed the services. Prevalence could also be higher among those who miss their PCR results. A critical analysis of the factors underlying the persistently high HIV prevalence among exposed infants despite increasing availability of PMTCT services will inform further targeted efforts towards eMTCT targets. We sought to determine the prevalence and factors associated with HIV positive sero-status among HIV exposed infants in Kasese district in Uganda. The study also explored the health systems factors affecting eMTCT service delivery in the district.

## Methods

### Study setting

The study was done in ten health facilities in Kasese district, including Bwera hospital, Kasese Municipal HC III, Kilembe Mines hospital, Kasanga PHC HC III, St. Paul HC IV, Hima government HC III, Rukoki HC III, Kagando hospital, Katwe HC III and Bishop Masereka Christian Foundation HC IV. These sites were selected because they contributed 76% of exposed infants in 2013/2014. Option B+ was rolled out at these facilities in March 2013 as a strategy for eMTCT. Pregnant and lactating mothers who tested HIV positive were initiated on long term ART for their own health and for the benefit of their exposed babies. After delivery, exposed babies were initiated on Neverapine syrup prophylaxis for six weeks. The first PCR test for the baby was scheduled for six weeks thus after completion of the Neverapine prophylaxis.

### HIV testing of study participants

The main entry point for eMTCT services is Provider Initiated Counselling and Testing (PICT) during antenatal care. Serological HIV test for mothers was done using Determine™ HIV-1/2 (Alere Medical Company Limited, Chiba, Japan), HIV 1/2 Stat-Pak^R^ Dipstick (Chembio Diagnostic Systems, Medford, NY - USA) and Uni-Gold™ HIV (Trinity Biotech, Bray, Ireland). Those who missed antenatal care were tested during delivery, labour and post-natal. Pregnant and lactating mothers who tested HIV positive were initiated on ART. After delivery, exposed babies were initiated on Neverapine syrup prophylaxis for six weeks. This time also coincided with the first PCR test for the baby. The second PCR was done six weeks after cessation of breastfeeding. Blood samples were collected using Dry Blood Spot (DBS) filter papers manufactured by Schleicher & Schuell Bioscience, Inc. These were transported to Kampala where a DNA/PCR was done at Central Public Health Laboratory (CPHL) using COBAS® AmpliPrep/COBAS® TaqMan**®** HIV-1 Test, v2.0. HIV test results were sent back through post office to the health facility laboratory. HIV exposed infants were followed up until 18 months of age when their HIV status was determined using serological test. Those testing positive at any stage of care continuum were enrolled for care and treatment at the ART clinic. Confirmed HIV negative children were discharged from care while their mothers continued receiving treatment at the ART clinic.

### Study design and data collection procedures

This was a cross-sectional study using mixed methods. The study was conducted between April and June 2016.

### Quantitative data

Data about the mother was extracted from the comprehensive HIV care card. This included: WHO clinical staging at enrolment, CD4 count at enrollment, ARV regimen (1^st^ line or second line), ART treatment interruptions, linkage to home based care, adherence to treatment and whether or not a mother was attached to a treatment supporter/buddy. Poor adherence was defined as taking ≤85% of the prescribed medicine and missing >5 doses of a one daily dosing or missing >9 doses of a 2-daily dosing per month [12]. Infant data was extracted from the exposed infant clinical charts and this included: age in months of enrollment at mother baby care point, date of birth, age at Neverapine syrup prophylaxis, receipt of infant ARVs for prophylaxis, baby HIV testing information, breast feeding option for the baby, place of birth, mode of delivery, mother’s ARVs during antenatal, labour and postpartum.

### Inclusion criteria

All HIV exposed infants who were 18 months of age and above and their mothers were included in the study. These were enrolled at mother-baby care point from March 2013 when eMTCT was first implemented in Kasese district to April 2016 when data was collected.

### Qualitative data

Two health workers including the PMCT and EID focal persons from each of the ten participating facilities were purposively interviewed as key informants (20 Health workers over all). Two mothers who were active in care from each health facility were conveniently selected for in-depth interviews. Seven in-depth interviews were conducted with mothers of exposed infants who had not come to the health facility for three months or more to explore their experiences and challenges. These were identified from the appointment register. Mothers were interviewed through home follow up visits by either a peer mother, VHT or an expert client. Key informant and in-depth interview guides were used to interview health workers and mothers respectively.

### Quality control

Research assistants were trained on the research protocol and data collection tools before start of data collection. The study tools were pre-tested at Kasese Municipal HC III within Kasese town. After data collection, each completed tool was checked for completeness, accuracy and consistency and incomplete tools were given back to the research assistant for completion with reference to the routine patient records. Where information was not available on any of the source document, it was reported as not documented.

### Measures

HIV status of the baby (positive or negative) was the main outcome variable. HIV status was determined using results from the 1^st^ PCR and 2^nd^ PCR tests as well as rapid HIV test at 18 months of age. The independent variables were categorized as maternal and infant factors. Maternal factors included receipt of ART, active in care/retention in care (continuous engagement in HIV care and treatment services and mother not out of care for >3moths), adherence to clinic appointments, place of delivery, mode of delivery, disclosure of HIV status to significant others (sexual partner, family member or friend), adherence to ART measured as the number of pills taken out of pills dispensed X100, CD4 count, WHO clinical stage at enrollment, mother attachment to treatment buddy and level of health facility. Infant factors included age at NVP prophylaxis, age of enrollment at baby care point and baby feeding options. Breastfeeding information was available on the routine data collection tool. To determine health systems factors affecting implementation of eMTCT services, the following qualitative themes were explored: perspectives on quality of eMTCT services, availability of eMTCT supplies, human resources for eMTCT, community follow up system for mother-baby pairs, social support groups, reasons for failing to meet clinic appointments and challenges experienced while seeking care and treatment.

### Data analysis

Quantitative data was entered in Epi Info version 3.5.4 and analyzed using Stata version 12. Descriptive statistics was used to describe infant and mother characteristics. The measures included proportions for categorical variable, percentiles and range for continuous variable like age. Age for both infant and mother were categorized before generating percentiles.

The primary outcome variable was categorized as “0” for HIV negative and “1” for HIV positive. Prevalence among exposed infants was calculated as the number of HIV positive infants divided by the total number exposed who received their final HIV test results. Final infant HIV status was determined by either a PCR or rapid HIV test as appropriate to infants’ age and breastfeeding option. The HIV test was considered final if the PCR was done before 18 months of age but six weeks after cessation of breast feeding or a rapid test done at 18 months of age but at least six weeks after stopping breast feeding. Data was modeled using logistic regression to evaluate factors associated with HIV sero-status among exposed babies attending mother-baby care points at selected health facilities in Kasese district. At bivariate level, HIV final status was analysed against all the independent variables and the resulting Odds ratios used as measures of association. All independent variables with a P-value of <0.2 at bivariate level were entered into a full logistic regression model. Multivariable logistic regression modelling strategy was used to evaluate confounding and develop a final model. Any variable with a P-value of <0.05 was considered as statistically significant.

Health care workers were interviewed as key informants and in-depth interviews conducted with mothers of HIV exposed infants. Latent content analysis was used to manually analyse qualitative data. Key themes were categorized and responses coded along apriori health system factors affecting HIV prevention interventions among exposed infants attending care at health facilities in Kasese district.

## Results

### Quantitative findings

Data analysis included 647 mother-baby pairs from all the participating ten health facilities. These included 530/647 (82%) mothers whose age was documented. Most of them; 294/530 (56%) were in the age category of 25–34 and 194/530 (37%) in the age group 16 – 24 years at enrollment into HIV care and treatment. Overall, 580/647 (90%) were ART naïve and 409/595 (69%) in WHO stage I. Those who received a CD4 count at enrollment into eMTCT services were 73/647 (12.1%) with average CD4 of 473. About 334/647(51.6%) were enrolled at health centre III, 64/647 (9.9%) at health centre IV and 249/647 (38.5%) at the three study hospitals. At the time of ART initiation, 381/647 (59%) were pregnant and 122/647 (19%) lactating. Pregnancy status was not documented for 116/647 (18%) while 28/647 (4%) initiated ART for their own health before pregnancy. The majority 456/647 (70.5%) were married while 57/647 (8.8%) were separated or divorced and 60/647(9.3%) were single.

Majority of the infants 460/634 (73%) enrolled at mother-baby care point when they were one to six months of age while 101/634 (16%) were less than one month. The female infants were 347/646 (54%). About 468/647 (72.3%) of all infants were delivered at either a public or PNFP health facility. Delivery for 73/647 (11.3%) of the infants was outside health facilities while 49/647 (7.6%) were delivered at private for profit clinics and the place of birth was not documented for 57/647(8.8%) (Table 1).

**Table 1:** Background characteristics of study participants

Variables	Frequency (N=647)	%
Mother		
Age in years^a^		
≤15 years	3	0.6
16 – 24	194	36.6
25 -34	294	55.5
35 – 44	37	7.0
≥45	2	0.4
Marital status		
Married	456	70.5
Separated/Divorced	57	8.8
Single	60	9.3
Widowed	12	1.9
Not documented	62	9.6
Facility level		
HC III	334	51.6
HC IV	64	9.9
Hospital	249	38.5
Entry care point		
IPD	19	2.9
MCH	307	47.5
Maternity	27	4.2
OPD	167	25.8
Not documented	62	9.6
Others	65	10.1
Use of ART before pregnancy		
Yes	67	10.4
No	580	89.6
WHO Stage at enrollment^a^		
I	409	68.7
II	121	20.3
III	62	10.4
IV	3	0.5
CD4 at enrolment		
Average CD4 (78 Observations )	473	NA
Pregnancy status at ART start		
Pregnant	381	58.9
Lactating	122	18.9
None	28	4.3
Not documented	116	17.9
Infant characteristics		
Age at registration (in months)^a^		
<1 month	101	15.9
1 – 6 months	460	72.6
7 – 12 months	42	6.6
13 – 18 months	22	3.5
≥19 months	9	1.4
Sex^a^		
Female	347	53.7
Male	299	46.3
Place of delivery		
Health facility-Public & PNFP	468	72.3
Outside health facility	73	11.3
Private clinics	49	7.6
Unknown	57	8.8
Mode of delivery		
Cesarean section	81	12.5
Normal vaginal delivery	496	76.7
Vacuum delivery	2	0.3
Not documented	68	10.5

^a^N-less than 647 because of missing values during data collection

### HIV Prevalence among exposed infants

Of the 647 exposed infants in the study, 493 (76%) had their HIV status determined and 32 (6.5%) were HIV positive. Those whose HIV test outcome was not determined did not return to the clinic to have the test done. The highest prevalence was registered at Kagando hospital 3/22 (13.6%) and Katwe HC III 2/17 (11.8%). Prevalence was highest among infants delivered at private clinics 5/34 (14.7%) and outside health facilities 5/54 (9.3%) as shown in Table 2.

**Table 2:** HIV prevalence among exposed infants

Variables	*N* = 493	HIV+ve (n)	Prevalence
Overall prevalence	493	32	6.5
Name of health facility			
Bishop Masereka HC III	30	1	3.3
Bwera Hospital	111	4	3.6
Hima HC III	43	5	11.6
Kagando Hospital	22	3	13.6
Kasanga PHC	54	5	9.3
Kasese Town Council	72	2	2.8
Katwe HC III	17	2	11.8
Kilembe Mines Hospital	60	2	3.3
Rukoki HC III	39	3	7.7
St.Paul HC IV	45	5	11.1
Marital status			
Married	347	17	4.9
Separated/Divorced	45	4	8.9
Single	47	7	14.9
Widowed	9	0	0.0
Not documented	45	4	8.9
Entry care point			
IPD	16	1	6.3
MCH	224	9	6.3
Maternity	22	1	4.6
OPD	136	9	6.6
Not documented	50	8	16.0
Others	45	4	8.9
Level of facility			
HC III	254	18	7.1
HC IV	47	5	10.6
Hospital	192	9	4.7
Place of delivery			
Health facility-Public & PNFP	368	11	3.0
Outside health facility	54	5	9.3
Private clinics	34	5	14.7
Caretaker did not know	37	11	29.7
Mode of delivery			
Cesarean section	67	0	0.0
Normal virginal delivery	372	24	6.5
Vacuum delivery	2	1	50.0
Not documented	52	7	13.5

### Factors associated with HIV positive sero-status

#### Maternal factors

At bivariate analysis, mothers who had poor adherence were three times more likely to have an HIV positive baby (OR = 3.23, 95% CI: 1.013–10.304, *p* = 0.047). Infants of mothers who were either lost from care or dead were four times more likely to be HIV positive (OR = 3.6, 95% CI: 1.265 - 10.508, *p* = 0.017). The place of delivery was associated with HIV positive status. Babies who were delivered at private clinics were six times more likely to be HIV positive (OR = 5.6, 95% CI: 1.104 - 9.933, *p* = 0.003). Infants who were delivered outside the health facility were seven times more likely to be HIV positive than those delivered in health facilities (OR = 6.9, 95% CI = 3.089–15.517, *p* <0.001). Infants whose mothers delivered through vacuum extraction were six times likely to be HIV positive than those delivered through spontaneous normal vaginal delivery (OR = 6.4, 95% CI: 0.36 – 114.97, *p* = 0.206). Mothers who never kept clinic appointments were 2 times more likely to have a positive baby than those who kept appointment (OR = 1.5, 95% CI: 0.663 – 3.782, *p* = 0.3) as depicted in Table 3.

**Table 3:** Unadjusted and adjusted maternal factors associated with HIV positive sero-status

Characteristics	Number	Positive	Un adjusted OR (95% CI)	*P*-value	Adjusted OR (95% CI)	*P*-value AOR
Place of delivery						
Health facility-Public & PNFP	368	11	1			
Outside health facility	91	16	6.9(3.089-15.517)	<0.001*	5.1(1.038-24.742)	0.045*
Private clinics	34	5	5.9(1.820-17.198)	0.003		
ART Adherence^a^						
Good	367	18	1			
Fair	28	1	0.72(0.092 - 5.585)	0.752		
Poor	28	4	3.23(1.013-10.304)	0.047	4.5(0.411-49.398)	0.218
WHO staging at enrollment^a^						
I	311	19	1			
II	94	7	1.23(0.503-3.038)	0.64		
III	44	3	1.12(0.319-3.97)	0.85		
IV	2	0				
Gestation age at ART/prophylaxis^a^						
1st trimester:1 – 12weeks	26	1	1			
2nd trimester:13 – 28weeks	78	1	0.3(0.019-5.383)	0.432		
3rd trimester: 29 -40weeks	35	2	1.5(0.129-17.664)	0.74		
Duration on ART before delivery						
>6 months	155	1	1			
<6 months	192	7	5.7 (0.688 - 46.423)	0.107	4.1(0.471-35.775)	0.201
Mother care status at child final testing						
Active on treatment /Referrals	391	22	1		1	
Dead/Lost	28	5	3.6 (1.265 - 10.508)	0.017*		
Missed appointment/Stopped treatment	41	2	0.8 (0.194 - 3.796)	0.842	2.4(0.401-14.448)	0.336
Information not documented	30	3	1.7 (0.474 - 5.927)	0.422		
Appointments kept^a^						
Yes	347	17	1			
No	106	8	1.5 (0.663 - 3.782)	0.300		
CD4^a^						
<350	38	2	1			
350 - 499	12	1	1.6 (0.135 - 19.808)	0.699		
>500 cells	25	3	2.5 (0.379 - 15.864)	0.346		
Treatment supporter^a^						
Yes	360	24	1			
No	57	1	0.3 (0.033 - 1.885)	0.179		
Not documented	48	2	0.6 (0.139 - 2.66.0)	0.509		
Linkage to home based care						
Yes	179	12	1			
No	111	5	0.65 (0.224 - 1.916)	0.441		
Not documented	179	11	0.9 (0.391 - 2.122)	0.829		

*p<0.05, ^a^N less than 493 because of missing values during data collection

#### Infant factors

At bivariate analysis, the infant’s age at registration into mother-baby care point was associated with HIV positive sero-status. Those who registered at 7-12 months of age were 9 times more likely to be HIV positive than those who registered early (OR = 8.7, 95% CI: 3.527 – 21.476 4, *p* < 0.001). Infants who registered at the age of ≥13 months were 8 times more likely to be HIV positive (OR = 8.1, 95% CI: 2.838 – 22.880, *p* <0.001) and infants who did not receive any form of prophylaxis were nine times more likely to be HIV positive (OR=9.1, 95% CI: 3.904 – 21.173, *p* <0.001) than those who received prophylaxis. Infants who were mixed fed were 10 times more likely to be HIV positive (OR = 9.5, 95% CI: 3.904 – 21.173, *p* = 0.002) as described in Table 4.

**Table 4:** Unadjusted and adjusted infant factors associated with HIV positive sero-status

Characteristics	Number	Positive	Unadjusted OR (95% CI)	*P*-value	Adjusted OR (95% CI)	*P*-value AOR
Age at registration (in months)						
<7months	428	17	1		1	
7- 12 months	34	9	8.7 (3.527 - 21.476)	<0.001	2.5 (0.704 – 9.140)	0.154
>13 months	24	6	8.1(2.838 - 22.880)	<0.001*	2.9 (0.772 – 11.153)	0.114
Not documented	7	0				
Infant ARVs for prophylaxis						
Received any form of ART at birth	370	9	1		1	
No ARVs at birth	92	17	9.1(3.904 – 21.173)	<0.001*	4.9 (1.901 – 13.051)	0.001*
Unknown	24	4	8.0 (2.273 – 28.307)	0.001*	4.8 (1.264 – 18.580)	0.021*
Not documented	7	2	16.0 (2.738 – 94.024)	0.002*	11.7 (1.753 –77.689	0.011*
Feeding methods at registration						
Exclusive breast feeding	425	16	1		1	
Replacement feeding	5	1	6.3 (0.675 - 60. 481)	0.106	7.9 (0.733 – 87.0187)	0.088
Mixed feeding	11	3	9.5 (2.322 - 39.570)	0.002*	3.4 (0.639 – 18.170)	0.151
Complimentary feeding	41	10	8.2 (3.453 - 19.689)	0.000*	1.8 (0. 511- 6.329)	0.36
No longer breast feeding (NLB)	9	2	7.3 (1.404 - 37.989)	0.018*	2.6 (0.031 - 0.412)	0.311
Not documented	2	0				

*p<0.05

At multivariate analysis, two factors remained statistically significant. Mothers who delivered outside the health facility were five times more likely to have an HIV positive baby (AOR = 5.1, 95% CI: 1.038 – 24.742, *p* = 0.045). Infants who never received ARVs for prophylaxis at birth were 5 times more likely to test HIV positive (AOR = 4.9, 95% CI: 1.901–13.051, *p* = 0.001). The details are shown in Tables 3 and 4.

### Qualitative findings on health system factors affecting implementation of eMTCT services

#### Qualitative findings were derived from 20 KI interviews and 27 in-depth interviews (Table 5)

**Table 5:** Categories and number of interviews conducted

Methods	Category of participants	Number of interviews	Total number of participants
Key informant Interviews	Health workers at mother baby care point	2 health workers for each facility	20
In-depth interviews	Mothers of exposed infants receiving care and treatment at 10 health facilities	2 mothers per facility for those in care and 1 mother among those who missed clinic visits	27 mothers (20 active in care and 7 missed clinic appoints for ≥ 3months)

##### Key informant interview with health workers

KI respondents reported several aspects of the eMTCT services as going on well across the health facilities in the study area including: enrollment of HIV positive mothers in care and treatment services, eMTCT services were done on daily basis, health education talks on option B+ package were provided, testing almost all mothers at ANC for HIV, giving follow up appointments to mothers and their exposed infants, testing exposed infants for DNA/PCR, reduced HIV infection among babies, steady supply of ARVS, counseling of mothers, syphilis testing for mothers/partners and availability of health workers. “*eMTCT is working well. The number of children testing HIV Positive are reducing steadily” (KI_St.Paul HC IV).*

However, they mentioned several areas that need improvement. The health workers cited a number of issues that were not working well including: few mothers bringing their infants for the 2^nd^ PCR test, understaffing of the ART clinics, challenges with tracking of women and infants who miss appointments, undocumented self-referrals, incomplete locator information, poor telephone network coverage, lack of transport for follow up and competing duties at the facility. More so, Family Support Groups (FSGs) were not well facilitated and yet they are crucial in bringing families of mothers living with HIV together which ultimately improves retention in care and treatment services. It was also a common practice for mothers to deliver at home which affected implementation of eMTCT services.

*What is not working well is that we are understaffed. So there is work overload* (*KI_ Rukoki HC III*).

*If mothers come when they have already delivered and not in the programme, some children are turning HIV positive on 1*^*st*^ *PCR* (*KI_Bwera hospital*).

*Family support meetings; not going on well. Mothers do not come as scheduled in the morning; others come early, others late* (*KI_II, Rukoki HC III*).

##### Management of eMTCT supplies

All key informants reported improvement in access to eMTCT supplies including ARVs and HIV testing commodities. “*We are well stocked and I cannot recall since we last had a stock out” (KI_Kilembe hospital).* However, some KIs reported stock out of Neverapine oral suspension, TDF/3TC/EFV, viral load books, DBS filter papers and HIV test kits. During shortages, health facilities borrowed from other nearby facilities and placed emergency orders to the HIV implementing partners. “*We always borrow from another facility in case medical access has not responded to the report submitted” (KI_St.Paul HC IV).* Most of the health workers knew how regular orders were submitted every two months.

##### Human resources for eMTCT

Staffing level was mentioned as a challenge to implementation of eMTCT services by most of the KIs. Most sites had about two staff allocated to mother-baby care points and staff mentioned this was not adequate since they had other duties assigned besides care and treatment for mothers and their exposed infants.

*We have very limited staff working on exposed infants and yet we are required to provide other services. At times you are allocated on the medical ward and when a mother of an exposed infant comes, you are expected to attend to her* (*KI_St.Paul HC IV*).

*Not well staffed. I work at EID alone. Does bleeding for viral load, CD4 sample collection, DBS collection and yet the lab would have a role in sample collection* (*KI_Bishop Masereka Medical Centre*)*.*

##### Community follow up system for mother-baby pairs who miss clinic appointments

The health facilities identified clients who did not keep appointments through use of appointment books, use of registers especially exposed infant registers and open MRS (medical record system). They tracked those who were lost through telephone calls, use of Village Health Teams (VHTs), FSGs and other community structures. To improve the community follow up system, health workers suggested that clients should provide right addresses, implementing partners and the district should facilitate home visiting of clients, provide airtime for follow-up, involve the administrators in eMTCT activities and provide motorcycles for follow up activities.

*LCs and VHTs should be sensitized and facilitated to support eMTCT services* (*KI_Rukoki HC III*).

*There is need for motorcycles for health workers to do follow up on missed appointments or transport for follow up* (*KI_Kilembe hospital*).

##### In-depth interviews with mothers of exposed infants

The mothers reported receiving the following services from health facilities: drugs which included ARVs for mothers, Septrin for adults, Septrin and ARV syrup for the baby, blood testing for CD4, water vessels and insecticide treated mosquito nets. They appreciated that ARVs were never out of stock at health facilities, the care received from health workers, privacy and confidentiality

*The service is good and I like it because I was well counseled and the councilors especially Janet and she gave me guidelines to follow while taking drugs. The Challenge is that I don’t know my blood group (IDI_Bishop Masereka Medical)*.

*The services at this health facility are good because drugs are always available and health workers care about the patients (IDI _Hima HC III)*.

However, HIV positive mothers did not like long waiting time when they come for appointments, Septrin stock out, user charges for laboratory tests and drugs for opportunistic infections by Private Not for Profit (PNFPs) facilities and disclosure of their HIV positive status to other people.

*The services at this health centre are fine however, the challenge is that sometimes we lack Septrin (IDI _Kasese Municipal HC III)*.

*People who knew me who come to that health facility were too many and I felt they would discover my status (IDI _Bishop Masereka Medical***)**.

Most mothers chose the health facility for care and treatment because of ease access from their area of residence. Other reasons for choosing the health facility were: when mothers felt their HIV status would be kept confidential by health workers at a particular health facility, being a government health facility with free services, testing HIV positive during antenatal services, being brought by a friend and feeling that health workers at a particular clinic were caring.

*I chose Hima Health facility because it’s where I tested positive and it’s near my home (IDI_Hima HC III)*.

*Because it is a government health facility and we get free drugs supplied by the government (IDI_Kasese municipal council HC III)*.

##### Reasons for failing to come to health facility

A number of reasons were given by mothers for failure to come to the health facility and these were: when visited by family members on days scheduled for clinic appointment and they fear inadvertent disclosure by going to the health facility in presence of a relative, lack of transport to health facility, sickness leading to admission to another health facility, not allowed by employers to go to facilities for health care, travel to visit relatives, forgetting appointment dates and domestic violence.

*I failed to pick medicines when I was very sick and later on I was admitted to Kagando hospital which is near my parents. I now get drugs from Kagando hospital but I may come to Kasese when I get better (IDI_Kasese Municipal HC III)*.

*I am working as a domestic worker and it is hard to leave and come for treatment (IDI_Bishop Masereka Medical Centre).*

*Domestic violence made me divorce and I moved far away from the health facility (KI_MunicipalHC III)*.

##### Reasons for being out of care and treatment for three months or more

The mothers of HIV exposed infants said they had not come to the health facility in three months because of: care for sick family member, misunderstanding with husband who was HIV negative (Discordant couple), change of employment and relocated to another area, went home to give birth at her mother’s home and domestic violence which led to separation and change of residence. In addition, 4/7 of the mothers who had not reported for treatment had enrolled in another health facility.

*I got a job in Kamwenge and relocated. I couldn’t manage monthly visits from Kamwenge to Kasese. I am however getting treatment from Padre pio HC III (IDI_ Bishop Masereka Medical Centre)*.

*I had gone home to give birth and get care from my mother. I will be coming soon to the hospital (IDI_Bwera hospital).*

##### Mothers’ perceptions of the quality of services

The opinion of mothers on quality of services was sought and 22/27 rated the services as good, 2/27 rated them as fair, 1/27 as excellent, 1/27 said quality of services had declined and 1/27 said the quality was poor.

*The services are good but the problem is with me the client who had failed to come to the facility* (*IDI_ Bishop Masereka Medical Centre)*.

*The services are fair because sometimes drugs for other diseases like malaria, cough do get out of stock then we buy from drug shops (IDI_RukokiHC III)*.

*Services are fair but time management by health workers is poor. They attend to patients up to late in the evening* (*IDI_Bwera Hospital*).

The challenges faced by mothers of exposed infants while seeking care and treatment services were: lack of transport to come for clinic appointments, poor time management by health workers during clinic visits, buying drugs that were out of stock and user charges by PNFP health facilities.

*We spend long time at the health facility without getting treatment while feeling hungry* (*IDI_Kasese Municipal HC III*).

*They ask for money and yet I was never asked for money at Bwera hospital while I was attending there* (*IDI_St Paul HC IV*).

To address the above challenges, mothers suggested a number of interventions and these were: transfer to a nearby treatment centre to solve the transport problem, train health workers on time management, hire more health workers, delegation of duties among health workers, provide all medicines for HIV positive clients, health workers should avoid disclosing their HIV positive status to other people and attach clients to treatment supporters.

*Health workers should be trained on time management since some clients miss taking drugs on the ART clinic day* (IDI_Bwera Hospital).

*Government should recruit enough health workers to help those seeking care and treatment at all government facilities* (*IDI_Municipal HC III*).

## Discussion

Despite the availability of PMTCT services in Uganda since 2000, the country is still challenged with a high burden of paediatric HIV. In this study, we found that HIV prevalence among HIV exposed infants was 6.5%. The factors associated with HIV positive status of the infant included the delivery out of health facilities and failure to initiate infants on ART prophylaxis at birth. At 6.5%, the overall HIV prevalence among exposed infants remains high and above the eMTCT targets, although this represents a significant decline from 8% in 2011 [8]. The HIV prevalence is also slightly higher than the 5.3% estimated national level prevalence among exposed infants [10, 11]. The difference could be due to the methods used in calculating prevalence. The Ministry of Health findings were based on 1^st^ PCR results which may not provide a correct estimate of HIV prevalence among exposed infants. The high prevalence could also be due to the presence of many key populations in Kasese district [13, 14].

At multivariate analysis, factors associated with HIV positive sero-status were failure to initiate the exposed infant on ART prophylaxis at birth and delivery outside health facilities. Similar studies by John Kinuthia in Kenya and Tariku Tadele in Ethiopia found none ARV prophylaxis significantly associated with infant HIV infection [15, 16]. The high prevalence among babies delivered in private clinics is suggestive of potential service quality challenges in these facilities and a need for support to bridge the gaps. A study in Nigeria identified lack of funding for PMTCT services at private and rural health facilities among the major barriers preventing the use of PMTCT services [17]. Dissatisfaction with antiretroviral services in private health facilities could threaten attainment of anti-retroviral treatment objectives [18]. Mothers should continously be educated about the challenges of assisted delivery by Traditional Birth Attendants [19]. None health facility delivery is associated with HIV infection among exposed infants [15]. The place of delivery especially home based was echoed by health workers among the factors affecting eMTCT services within Kasese district. A study in Ethiopia also found out that home delivery was significantly associated with maternal to child transmission of HIV [20]. Health workers will need to re-package counseling messages for the HIV positive mothers to understand the importance of post-natal care for their lives as well as for their exposed infants.

Adherence was not a significant factor at multivariate analysis most likely due to clients who leave pill balances at home and only bring empty containers leading to wrong classification of adherence levels. A study in South Africa identified that patients on ART were dumping medicines leading to over estimation of their adherence [21]. A previous study in Uganda found out high adherence levels determined by unannounced pill count and self-report compared with electronic adherence monitoring [22]. Infants of mothers who were either lost from care or dead were more likely to be HIV positive though not significant at multi variate analysis. Some of these children continued attending mother-baby care point with support from their grandmothers or close relatives and benefited from ART prophylaxis.

Infants who were mixed fed were likely to be HIV positive. However, this was not statistically significant at multi variate analysis. This contradicts studies that have shown that mixed breast feeding is significantly associated with HIV infection among HEIs [20]. The difference in this study may be because of the varying knowledge of health workers in classifying breast feeding options. A study in South African found out that only 6% of health workers could comprehensively explain the term “exclusive breastfeeding” as per WHO definition [23].

The human resource constraint came out strongly from both health workers and mothers of HIV exposed infants as a challenge affecting eMTCT services. This was consistent with findings by WHO [2]. Health workers should consider rescheduling mothers and giving long appointments especially those who are stable on treatment. The findings from this study show that, there was limited means of transport coupled with low and inconsistent funding. Whereas the MoH approach was giving bicycles to Village Health Teams (VHTs) for community follow up of clients, it was very limited especially in mountainous areas like Kasese. The MoH should instead consider an alternative means of motivating VHTs in mountainous arears rather than buying so many bicycles that will not support client home follow up. Stock outs for essential eMTCT supplies like Neverapine suspension and HIV test kits were quite common. This was consistent with a study by Baryamutuma et al about early assessment of Uganda’s roll-out of option B+ [24]. This was due to under delivery of these commodities from the national ware houses. Health facilities will need to regularly provide feedback to National Medical Stores on inadequate delivery of eMTCT supplies.

### Study limitations

This study was done in health facilities and therefore excluded HIV positive mothers and their exposed infants who were not enrolled into care. The calculated prevalence among HIV exposed infants could be an under-estimation of the population level prevalence among exposed infants since prevalence would likely be higher among those who are not enrolled in care. However, the findings still provide an opportunity for addressing the high HIV prevalence and identified factors among exposed babies in care. The data for this study was abstracted from health facility records and it had several gaps. More so, some of the files that were missing were excluded from the study.

## Conclusions

The prevalence of HIV among exposed infants attending care in Kasese district health facilities was still high and was associated with place of delivery and receipt of infant ARVs at birth. These were exacerbated by health system challenges especially staffing and a weak community follow up system for mother-baby pairs. Interventions to further reduce MTCT should focus on increasing facility based deliveries and ARVs for infants as well as relevant health and community programs to ensure support for women and their exposed infants.
